# 615. A Prospective Cohort Study to Evaluate Performance of Myco*MEIA – Aspergillus*: A New Urine Diagnostic for Aspergillosis

**DOI:** 10.1093/ofid/ofad500.681

**Published:** 2023-11-27

**Authors:** Nitipong Permpalung, Shakila Rajack, Kieren Marr, Kausik Datta, Dorothy MacLean-Blevins, Darin B Ostrander, Irina Baburina, Dana Neitzey, Patricia X Marques, Sean Zhang

**Affiliations:** Johns Hopkins University School of Medicine, Baltimore, MD; Johns Hopkins University School of Medicine, Baltimore, MD; John Hopkins, Bethesda, Maryland; Johns Hopkins University School of Medicine, Baltimore, MD; Johns Hopkins University School of Medicine, Baltimore, MD; Johns Hopkins University, Baltimore, Maryland; Pearl Diagnostics, Baltimore, Maryland; Pearl Diagnostics, Baltimore, Maryland; Pearl Diagnostics, Baltimore, Maryland; Johns Hopkins Hospital, Baltimore, Maryland

## Abstract

**Background:**

The Myco*MEIA* urine diagnostic was developed as an enzyme immunoassay, by Johns Hopkins (JH) and Pearl Diagnostics. In a retrospective study of 310 people, the assay was 90.5% (95% CI 70-99) sensitive and 89.2% (95% CI 82-94) specific. We report results of a prospective study performed at JH Hospital (JHH).

**Methods:**

People with suspected invasive aspergillosis (IA) were identified by screening of diagnostic tests sent by clinicians at JHH. Urines from consented subjects were tested in the clinical mycology lab within 8 hours of collection. Results were not used to inform care. Established diagnoses were adjudicated by clinicians blinded to lab results, using consensus criteria. Performance was analyzed in evaluable subjects who had CTs within 2 weeks of suspected IA. Findings supporting proven or probable IA, or no infection defined cases and controls, respectively. Myco*MEIA* results in subjects with possible IA, mixed infections, or other infections (non-IA) were evaluated descriptively.

**Results:**

107 urines were tested from 72 subjects with suspected IA. 15 subjects had >1 samples tested. Subjects were mostly male (50, 69%), with median age of 51 (range 7 – 87 years). IA was suspected during treatment for heme malignancies (59, 82%), solid organ transplant (9), cancer (5), and rheumatologic disease (1). Of 72 subjects enrolled, 30 had possible IA (42%), 30 no infection (42%), 4 probable IA (6%), 4 mixed infections (6%) and 4 other infections, including bacterial abscess (1), fusariosis (1), fungal sinusitis (1) and otitis externa (1). The assay was 100% sensitive (95% CI 76–98) and 93% specific (95% CI 79–99) for IA, with likelihood ratio of 15. Five of 30 (16.7%) people with possible IA and 1 with fusariosis had positive Myco*MEIA* urine tests.

**Conclusion:**

In prospective testing by the clinical lab, performance of the urine Myco*MEIA – Aspergillus* test appears consistent with retrospective findings and favorable compared to current blood assays. The proportion of lab confirmed diagnoses using current commercial assays was low; high negative predictive value with urine testing may support restriction of empirical antifungals. Cross reactivity amongst pathogenic Ascomycetes is biologically anticipated and requires further study. The study remains ongoing.

**Disclosures:**

**Nitipong Permpalung, MD, MPH**, Alcimed: Advisor/Consultant|CareDx: Grant/Research Support|Cidara Therapeutics: Grant/Research Support|Clarion: Advisor/Consultant|ClearView: Advisor/Consultant|IMMY Diagnostics: Grant/Research Support|Merck: Grant/Research Support|Scynexis: Grant/Research Support **Kieren Marr, MD**, Cidara Therapeutics: Advisor/Consultant|Merck: Advisor/Consultant|Pearl Diagnostics: salary|Pearl Diagnostics: Ownership Interest|Sfunga Therapeutics: salary|Sfunga Therapeutics: Ownership Interest **Irina Baburina, PhD**, Pearl Diagnostics: salary **Dana Neitzey, BS**, Pearl Diagnostics: salary **Patricia X. Marques, PhD**, Pearl Diagnostics: salary **Sean Zhang, MD, PhD**, Applied BioCode: Grant/Research Support|IMMY Diagnostics: Grant/Research Support|KARIUS: Advisor/Consultant|Pearl Diagnostics: Grant/Research Support|Scanogen: Grant/Research Support|T2 biosystems: Advisor/Consultant|Vela Diagnostics: Grant/Research Support

